# BNDB – The Biochemical Network Database

**DOI:** 10.1186/1471-2105-8-367

**Published:** 2007-10-02

**Authors:** Jan Küntzer, Christina Backes, Torsten Blum, Andreas Gerasch, Michael Kaufmann, Oliver Kohlbacher, Hans-Peter Lenhof

**Affiliations:** 1 Center for Bioinformatics, Saarland University, 66041 Saarbrücken, Germany; 2 Center for Bioinformatics/Wilhelm Schickard Institute for Computer Science, Eberhard Karls University Tübingen, 72076 Tübingen, Germany

## Abstract

**Background:**

Technological advances in high-throughput techniques and efficient data acquisition methods have resulted in a massive amount of life science data. The data is stored in numerous databases that have been established over the last decades and are essential resources for scientists nowadays. However, the diversity of the databases and the underlying data models make it difficult to combine this information for solving complex problems in systems biology. Currently, researchers typically have to browse several, often highly focused, databases to obtain the required information. Hence, there is a pressing need for more efficient systems for integrating, analyzing, and interpreting these data. The standardization and virtual consolidation of the databases is a major challenge resulting in a unified access to a variety of data sources.

**Description:**

We present the Biochemical Network Database (BNDB), a powerful relational database platform, allowing a complete semantic integration of an extensive collection of external databases. BNDB is built upon a comprehensive and extensible object model called BioCore, which is powerful enough to model most known biochemical processes and at the same time easily extensible to be adapted to new biological concepts. Besides a web interface for the search and curation of the data, a Java-based viewer (BiNA) provides a powerful platform-independent visualization and navigation of the data. BiNA uses sophisticated graph layout algorithms for an interactive visualization and navigation of BNDB.

**Conclusion:**

BNDB allows a simple, unified access to a variety of external data sources. Its tight integration with the biochemical network library BN++ offers the possibility for import, integration, analysis, and visualization of the data. BNDB is freely accessible at .

## Background

The development of high-throughput technologies has generated an extensive quantity of -omics data over the last decades. Despite the technological progress, improvements in the application area, e.g. in drug discovery, have failed to keep pace with increased research and development spending, as demonstrated by Nightingale et al. [1]. One of the main reasons for this discrepancy is the increasing number of highly focused databases differing in both the data models and the interfaces [2]. The databases are often independently developed, have a substantial overlap and are not well standardized. The absence of a standardization limits the usability of these databases and leads to a demand for a unified access to the data [3].

Hence, a large number of systems addressing this problem with diffierent approaches have been developed. These approaches can be classified by their architecture into three main categories [4]: *navigators*, *mediators*, and *warehouses*. The first category, navigators, is based on the idea of a navigational or link-based integration of several data sources. Such a portal normally does not integrate the data itself, but provides the user with pages navigating to external data sources. Well-established examples of portal systems are SRS [5], BioNavigator [6], and Entrez [7]. A mediator gives access to distributed data by reformulating the queries of the user at runtime into queries on external data sources. However, availability and efficiency are major drawbacks of such solutions. Examples for this category are Discovery Link [8], TAMBIS [9], and BioMediator [10]. Systems of the third category, warehouses, require a complete semantic integration of the data from various external data sources into a single local database via an integrative data model. Such approaches allow for an efficient execution of queries since they avoid typical problems of the other methods such as network bottlenecks, short-time unavailability of the external data sources, and changes in the external data sources. However, data warehouses usually require complex data models and regular updates of the integrated data sources, in order to avoid the possibility of returning outdated query results. BNDB is a representative of this category, as are other systems like GUS [11], ONDEX [12], cPath [13], and Biozon [14].

## Construction and content

**Figure 1:**
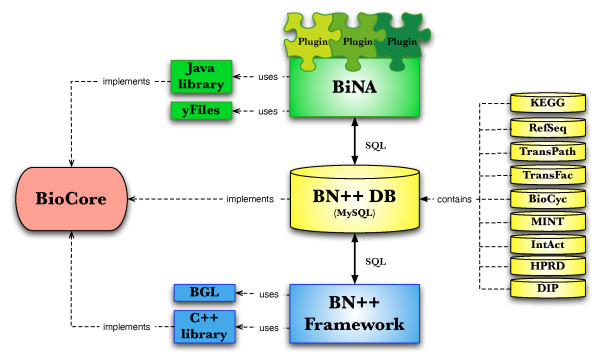
**Architecture**. Architecture of the BNDB data warehouse.

Based on an object-oriented data model, called BioCore, we developed and implemented BNDB, an SQL data warehouse system that integrates data sets from external and internal data sources via importers. The BioCore model allows not only for modelling of nearly all currently known biochemical processes, but also for including new biological concepts with little effort [15,16]. The architecture of the system is presented in Fig. 1.

**Figure 2:**
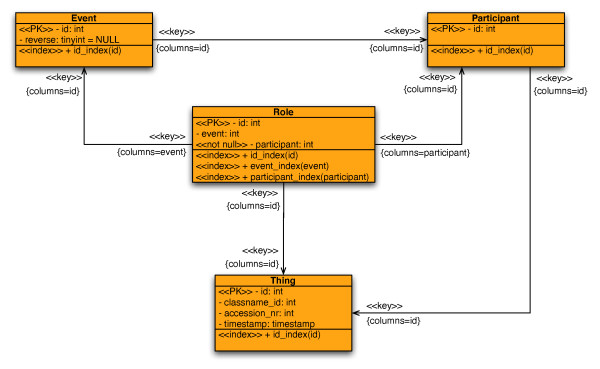
**Simplified DDL Diagram of BNDB**. The simplified structure of the database schema.

The BNDB is implemented as a relational database using MySQL [17]. We decided to chose a relational database management system over an object-oriented system, since relational DBMS are well-established and the current *de-facto *standard. This guarantees a high portability of the biochemical network database allowing a user to create a local version of the BNDB on a wide range of platforms. Therefore, we created an object-relational mapping of the BioCore model onto a relational database management system, using only SQL2 [18] compatible statements. This restriction allows the usage of any relational or object-relational database management system like DB2, Oracle, or PostgreSQL. The database consists of more than 240 tables representing all BioCore classes [see Additional file 1]. Additionally, BNDB includes tables for the user and rights management, as well as for the reconstruction of the object-oriented structure of the database. The schema for BNDB (Fig. 2) is available on the website.

In the current state, BNDB represents a comprehensive collection of biological data integrated from the following data sources:

• Sequence databases: SwissProt [19], RefSeq [20]

• Pathway databases: KEGG [21], BioCyc [22], TransPath [23]

• Protein interaction databases: DIP [24], MINT [25], IntAct [26], HPRD [27]

• Transcription factor databases: TransFac [28]

For the horizontal data integration [29,30] of these data we implemented comprehensive merging heuristics. The key concept behind these methods is the integration of complementary data sources and the elimination of redundancy in the data. We use two fundamental approaches for the merging of the data:

(1) object matching based on unambiguous external identifiers and (2) structural matching based on identical object relations.

**Figure 3:**
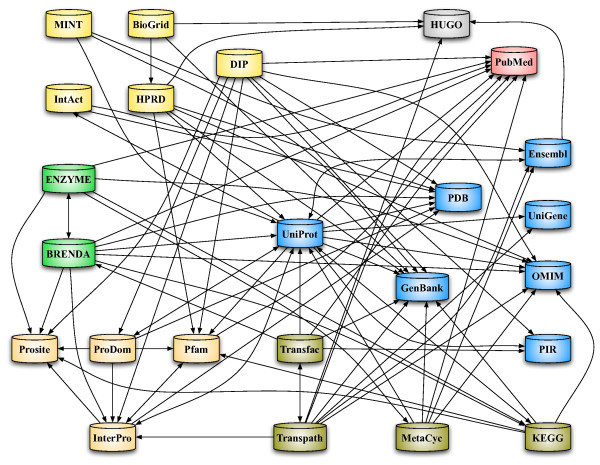
**Database Universe**. The nodes represent external databases labeled by their name. An edge is draw from A to B meaning that database A knows the ids of database B. In addition, the database are grouped by the contained data: the protein interaction dbs are yellow, enzyme dbs are green, the protein and sequence dbs are blue, pathway dbs are olive, and the orange nodes are domain dbs.

The first approach relies on the existence and correctness of selected standardized IDs in the imported databases (see Fig. 3). Each object in the database is linked with a variety of different external data source identifiers, like RefSeq, GeneId, SwissProt, Unigene, InterPro, etc. We only use those identifiers, that unambiguously identify the corresponding biochemical objects. For the merging we collect all unambiguous database identifiers in BNDB. For each of these IDs we check if they are connected to more than one object instance of the same type. If this is the case, we merge these instances into one single instance. All attributes of these instances are merged and multiple occurrences of these attributes are removed. External database IDs not describing unique objects, but rather clusters of objects (e.g. Unigene, InterPro, etc.) are not considered in the merging process. For objects without external identifiers, like biochemical events (e.g. metabolic reactions), we use the second approach based on structural matching of object relations. We define two events to be equal if they are of the same event type and contain the same participants occurring in the same role, whereas events, participants, and role are the major building blocks of the BioCore schema [15].

The merging process itself consists of several steps: In an initial step, we merge most of the database objects by their identifiers and remove redundancy in their attributes through the first approach. Then, in the second step we collect and merge all equivalent events in BNDB through the second structural approach.

**Figure 4:**
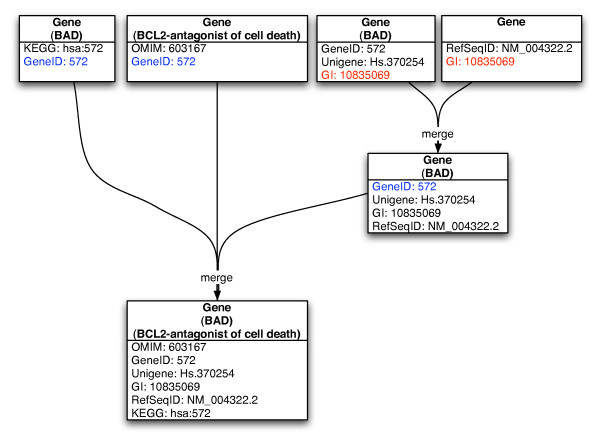
**Object matching based merging**. Simplified example for merging genes using the object matching based approach. In this case we have four instances of the human BAD gene, which we merge using the GI identifier and the GeneID. The resulting gene contains all merged names and identifiers.

A simplified example for merging genes using the first approach, is presented in Fig. 4. Four instances of the human BAD gene with different external database identifiers and names are merged by unambiguous identifiers. Two instances are connected with the same NCBI GI-ID and therefore identified to be equal. These instances are merged into one single instance connected with the merged attributes. The remaining instances are all associated with the same NCBI-GeneID. Thus, our algorithm merges these three instances into one single gene instance, which is linked with the merged information of all four former instances. All merging heuristics were implemented using the Biochemical Network Library BN++ [16] and the source code is available on our website.

## Utility and discussion

For accessing BNDB we offer three different ways: a web interface, a network visualizer, and a programming interface.

### Web interface

**Figure 5:**
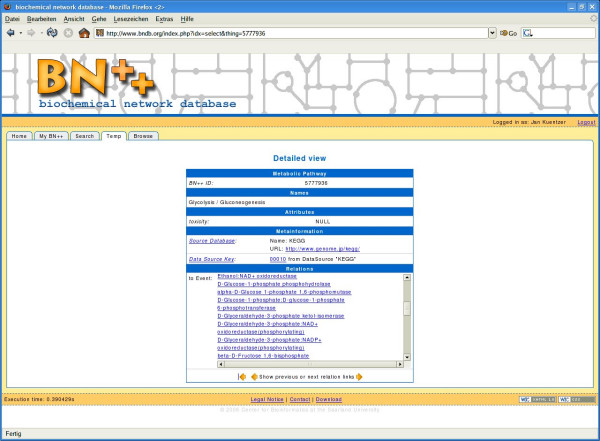
**Searching using the web interface**. Search for glycolysis in the web interface.

An intuitive web client browser enables querying and browsing BNDB. The user can search by name, description, or publication for participants, events and pathways. The user query is converted internally into an SQL query. For the standard search the user does not need to know any information about the internal structure of BNDB or its underlying data model BioCore. In addition, for more advanced users the web interface gives the possibility to perform direct SQL queries. The retrieved results are presented text- and link-based in a user-friendly way. Hyperlinks to external data sources are provided for additional information whenever external database identifiers are connected with the object (for an example see Fig. 5). Depending on the rights of the user, the system allows for a curation of the database by editing the displayed results. Furthermore, we included a functionality for adding new information in a convenient way, such that the user does not need to know the internal structure of the database. The BNDB interface guides the user through the adding process and warns if necessary information is missing.

### Network visualization

**Figure 6:**
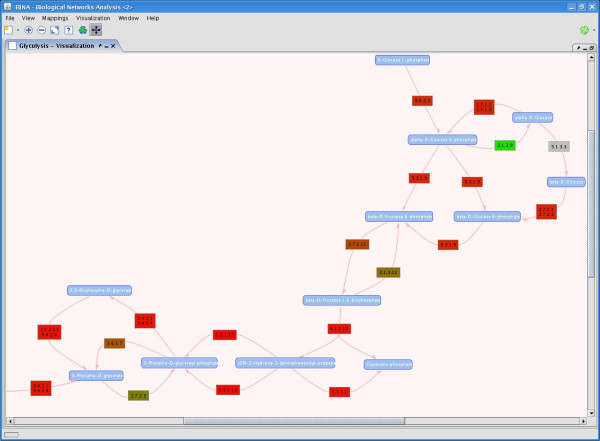
**Visualization using BiNA**. Visualization of the glycolysis using the metabolic graph layout. The blue boxes represent metabolic compounds. If there is an enzymatic reaction occurring between compounds, a directed edge labeled with the enzyme class catalyzing the reaction is drawn. The edge labels are colored by the expression value of the enzyme-coding genes. In this example we use expression values for the normal control of the GDS820 data set from the GEO database.

We provide a stand-alone Java application called BiNA for querying and analyzing the data contained in BNDB and for visualizing biological networks. The tool allows for visualizing metabolic and regulatory networks with sophisticated graph layout algorithms. Besides the direct visualization, BiNA also provides a mapping engine to analyze arbitrary data sets in the context of networks. This allows to map numerical biological data, e.g. mRNA expression data, onto graph attributes like node/edge color or size. The visualization of two data sets at the same time makes it easy to compare different data sets and identify correlations. The color scheme and the edge thickness used for the drawing can be freely defined by the user and is shown as a legend in the visualization view. Additionally, the mapped data values can be changed easily to interactively explore time-series expression data. In the metabolic view, the edges labeled with the catalyzing enzymes can be colored by the expression values of the enzyme-coding genes. In the regulatory view, the mapping plugin allows for coloring the nodes representing proteins, genes or protein families, whereas the protein families are colored by the values of all contained members. Fig. 6 gives an example for a metabolic view in BiNA with mapped expression data.

The graph and visualization capabilities of our application are comparable to that of visualization systems such as Cytoscape [31], PathSys [32], VisANT [33], or commercial tools such as MetaDrug [34] or PathwayStudio [35]. Additionally, BiNA offers a multifunctional workbench, which is easily extensible. The viewer itself can be regarded as a collection of modules that depend on each other. The hierarchical plugin system automatically resolves dependencies between plugins through a well-defined and very powerful interface. The plugin structure of BiNA allows for an easy integration of own analysis routines. Currently, several plugins exists, e.g. for mapping gene expression data onto the network, pathway search algorithms, or exporting pathways into SBML and BioPAX.

### Programming interface

BNDB is fully integrated with the Biochemical Network Library BN++ [15,16] providing a sophisticated programming interface. Hence, arbitrary data like a complete pathway can be serialized and deserialized from C++ by a single line of code. This speeds up the development process of analysis routines, since a programmer can concentrate on the implementation of the algorithm. In addition, the BN++ software framework offers a comprehensive collection of implemented analysis routines.

The C++ programming interface provides a convenient, but very flexible way to merge the data. With a few lines of code it is possible to construct a customized local meta-database containing only that data the user requires.

## Conclusion

With BNDB we present a data warehouse system integrating a large number of different biological databases. Access to these data is provided through a generic web interface allowing for adding, editing, and searching the data in BNDB. In addition, we have developed BiNA, a powerful and extensible tool for visualizing biochemical networks directly from BNDB. Through the BN++ software framework BNDB is easily accessible for software developers and can be integrated into tailor-made applications and customized to user needs. All tools and methods described herein, BNDB, BiNA, the source code, the web interface to BNDB, and the underlying data model are freely available from our website.

A major advantage of BNDB is its underlying data model BioCore. This comprehensive and extensible object model can represent most currently known biochemical entities and processes. Therefore, BNDB is able to store a huge variety of different biochemical data. Researchers can easily adapt it to their own needs and build customized databases. Another benefit is the full integration of BNDB into the visualizer BiNA. Other systems often present only a database with an analysis tool (e.g. Biozon), or a database with a web interface (e.g. Entrez). For the graphical representation of the networks, many of these systems use standard visualizer (e.g. Cytoscape). However, we think that the full integration of an own visualization tool facilitates the visualization and presentation of the stored data.

We have developed several applications based on BNDB that show the usefulness of the approach, e.g. an efficient gene set analysis tool, *GeneTrail *[36], which enables the user to identify enriched functional categories in protein or gene sets. *GeneTrail *has been successfully applied to detect a molecular target of the antimicrobial metabolite kendomycin [37].

In summary, BNDB is a comprehensive database system, which makes it not only possible to retrieve the combined information of integrated data sources in an easy way, but can also be customized and extended to meet the needs of different users.

## Availability and requirements

Project name: BNDB;

Project home page: ;

Operating system(s): Platform independent;

Programming language: Java; Other requirements: Java 1.6.0 or higher;

Licence: GNU GPL;

BNDB is freely accessible at . The current versions of BN++ and BiNA are distributed under the GNU GPL license and available from the website .

## Abbreviations

**BN++ **Biochemical Network Library

**BiNA **Biological Network Analysis

**DBMS **Database Management System

**NCBI **National Center for Biotechnology Information

**BGL **Boost Graph Library

**SQL **Standard Query Language

**SBML **Systems Biology Markup Language

**BioPAX **Biological Pathways Exchange

**GEO **Gene Expression Omnibus database

## Authors' contributions

AG programmed the network visualization tool. MK provided specialist knowledge on network visualization. JK, CB, and TB were involved in implementing one or more importers. JK, OK, and HPL contributed to the system design of BN++ and to the design of its data model. MK, OK and HPL supervised the project. All authors read and approved the final manuscript.

## Supplementary Material

Additional file 1 DDL Diagram of BNDB. The general structure of the database schema. Click here for file
